# Greeting

**DOI:** 10.31662/jmaj.s0001

**Published:** 2018-09-28

**Authors:** Yoshitake Yokokura

**Affiliations:** 1 Japan Medical Association

The Japan Medical Association (JMA) and the Japanese Association of Medical Sciences (JAMS) have launched the *JMA Journal*, a new official English medical journal.

Since the inception of the *Asian Medical Journal* in 1958 (renamed *JMAJ* in 2001), the JMA has been publishing Japanese medical studies and its activities in English.

With due consideration of the environment of global health and medical information provisions, the *JMA Journal*, a peer-reviewed publication, is issued with the cooperation of the JAMS as a new global platform for information dissemination, in place of the traditional *JMAJ* but continuing its traditions.

The *JMA Journal* covers all aspects of medicine and medical care, and includes contributions from medical researchers and healthcare professionals in Japan and elsewhere, on a wide range of areas, including original work, health policy, global health, and other commentary.

The publication of this type of comprehensive medical journal is the first challenge in Japan. We sincerely hope that the *JMA Journal* will promote international medical science and contribute to the improvement of the quality of health care by being read and cited by many people.

Yoshitake Yokokura, MD, PhD

President, Japan Medical Association

President, World Medical Association

